# Carboxylated dithiafulvenes and tetrathiafulvalene vinylogues: synthesis, electronic properties, and complexation with zinc ions

**DOI:** 10.3762/bjoc.11.107

**Published:** 2015-06-03

**Authors:** Yunfei Wang, Yuming Zhao

**Affiliations:** 1 Department of Chemistry, Memorial University, St. John’s, A1B 3X7, NL, Canada

**Keywords:** complexation, coordination polymers, porosity, redox activity, tetrathiafulvalene

## Abstract

A class of carboxyl and carboxylate ester-substituted dithiafulvene (DTF) derivatives and tetrathiafulvalene vinylogues (TTFVs) has been synthesized and their electronic and electrochemical redox properties were characterized by UV–vis spectroscopic and cyclic voltammetric analyses. The carboxyl-TTFV was applied as a redox-active ligand to complex with Zn(II) ions, forming a stable Zn-TTFV coordination polymer. The structural, electrochemical, and thermal properties of the coordination polymer were investigated by infrared spectroscopy, cyclic voltammetry, powder X-ray diffraction, and differential scanning calorimetric analyses. Furthermore, the microscopic porosity and surface area of the Zn-TTFV coordination polymer were measured by nitrogen gas adsorption analysis, showing a BET surface of 148.2 m^2^ g^−1^ and an average pore diameter of 10.2 nm.

## Introduction

**Scheme 1:**
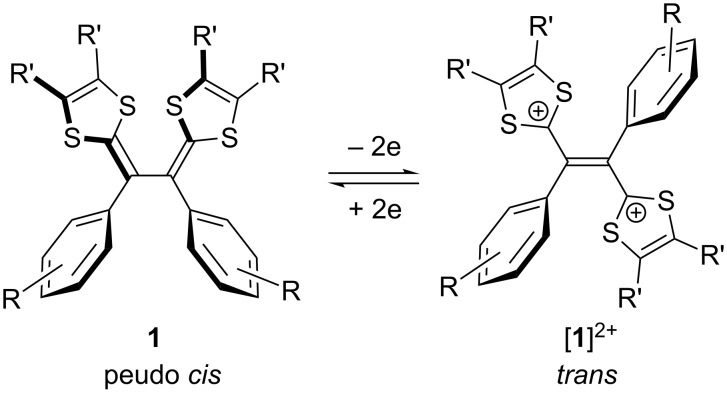
Redox-induced conformational switching of diphenyl-TTFV **1**.

Tetrathiafulvalene (TTF) has been widely applied as a redox-active building block in organic electronic materials and supramolecular assemblies [1–5], since the first discovery by Wudl and others in the early 1970s that TTF upon interactions with suitable electron acceptors could give rise to charge-transfer complexes exhibiting excellent metallic conductivity [6–7]. The remarkable electron-donating properties of TTF arise from its aromaticity-stabilized cationic states after releasing one and/or two electrons [1–5,8–10]. Tetrathiafulvalene vinylogues (TTFVs) are π-extended analogues of TTF bearing extended vinyl bridges between the two dithiole rings of TTF [9–11]. Similar to their parent TTF, TTFVs are excellent electron donors as well and they can undergo reversible electron transfers under mild redox conditions [11–15]. Of particular interest is the class of aryl-substituted TTFVs which show interesting conformational switching properties governed by redox processes [12,14–16]. For instance, the structure of diphenylated TTFV **1** can be transformed from a pseudo *cis* to a complete *trans* conformation upon oxidation (see Scheme 1). The application of TTFVs in material development began several decades ago, while the past few years have witnessed surging research activities on integrating TTFVs into a variety of π-conjugated molecular and macromolecular systems [12,17–26]. In many of the studies, the remarkable redox activity and intriguing conformational switching properties of TTFVs were taken advantage of to enhance structural and electronic properties as well as to introduce some “intelligent” functions such as conformational switchability and selectivity in terms of molecular recognition and supramolecular interactions.

**Scheme 2:**
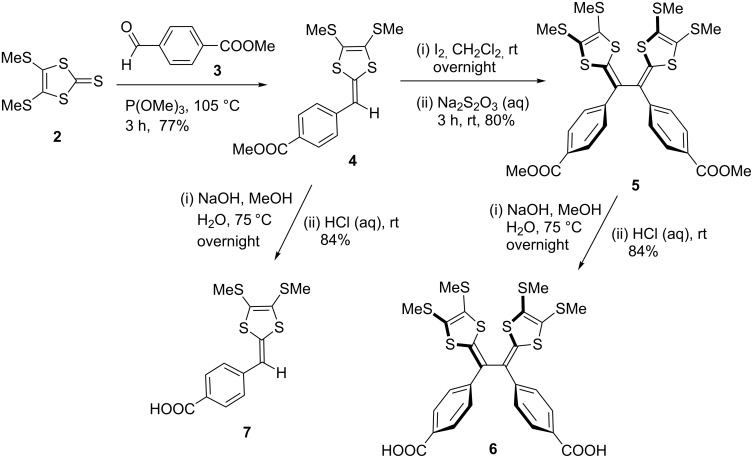
Synthesis of carboxylated TTFV **6** and DTF **7**.

The synthesis of TTFV structures is usually carried out through a facile iodine-promoted oxidative dimerization reaction of corresponding dithiafulvene (DTF) precursors [27]. This straightforward C–C bond forming reaction has not only allowed TTFV derivatives with different substituents to be readily assembled, but served as an effective methodology to construct the π-conjugated frameworks of some TTFV-based macrocycles and polymers [12,28]. Previously, we have investigated a series of diphenyl-TTFVs with alkynyl groups attached to the phenyl units as synthetic building blocks, through which extension of π-conjugated structures could be conveniently executed via the Pd-catalysed coupling and Cu-catalysed alkyne–azide cycloaddition (i.e., click) reactions [22–26]. In this work, we continued to explore the class of carboxylated diphenyl-TTFVs, in view of the synthetic versatility of the carboxyl group towards various commonly used linkage groups (e.g., amides, esters). The carboxyl group also presents a reliable and useful ligand to coordinate with transition metal ions, which in turn provides easy access to novel organic–inorganic hybrid materials. The most notable example of research in this context is the recent development of metal organic frameworks (MOFs), wherein the design and synthesis of carboxyl functional ligands has played a pivotal role prompting the advancement of this field [29–31]. Very recently, some TTF-based ligands have been employed to achieve organic–inorganic hybrid materials with redox activity [32–35]; however, the use of TTFVs as ligands has not been reported in the literature prior to this work. This article thus describes the first exploration of the synthesis and properties of a carboxylated diphenyl-TTFV **6** (Scheme 2) and its ability to form new redox-active porous materials through the formation of coordination polymer with Zn(II) ions.

## Results and Discussion

The synthesis of carboxyl-TTFV **6** and its DTF precursor **7** was conducted via a route well established for aryl-substituted TTFV derivatives [22–23]. As shown in Scheme 2, thione **2** was first reacted with benzaldehyde **3** in the presence of trimethylphosphite at 105 °C [36]. This olefination reaction went completion within 3 hours to give DTF **4** in 77% yield after column separation. Compound **4** was then subjected to an oxidative dimerization in CH_2_Cl_2_ at room temperature using iodine as oxidant. The dimerization gave TTFV **5** as a stable yellow solid in 80% yield. Saponification was then performed on compound **5** in a solution of NaOH in water and methanol to finally afford carboxyl-TTFV **6** in 84% yield. Compound **6** showed relatively poor solubility in non-polar organic solvents, but could be readily dissolved in polar solvents such as MeOH, EtOH, THF, and DMSO. For comparison purposes, carboxyl-DTF **7** was also prepared by hydrolysis of DTF **4** using similar reaction conditions.

**Scheme 3:**
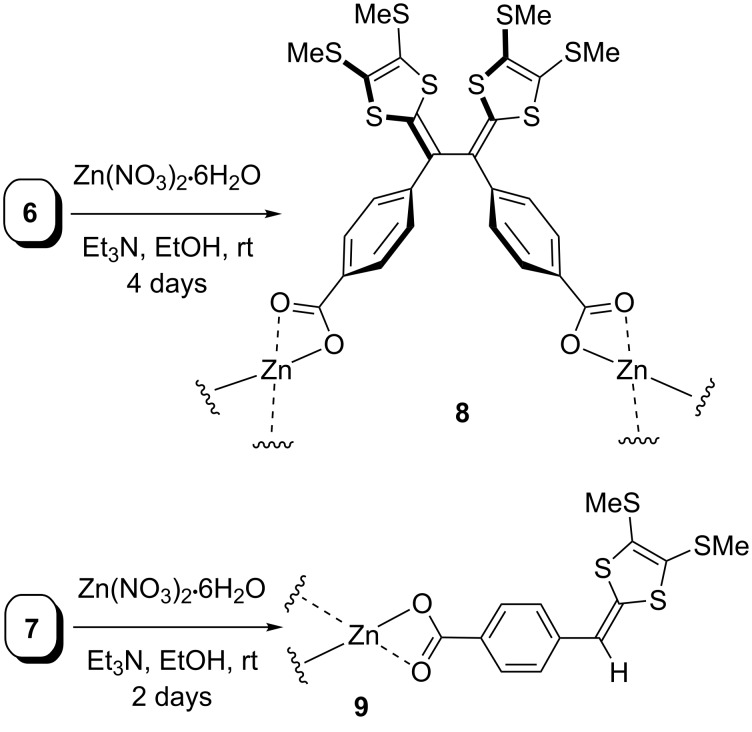
Complexation of compounds **6** and **7** with Zn(II) ions.

With carboxyl-TTFV **6** in hand, the preparation of coordination polymers with Zn(II) ions was undertaken. As outlined in Scheme 3, compound **6** was first mixed with two molar equivalents of Zn(NO_3_)_2_·6H_2_O in EtOH, and to this solution triethylamine was allowed to slowly diffuse in [37]. In a period of 4 days, coordination polymer **8** was gradually formed as a yellow coloured crystalline solid, which was insoluble in common solvents. By the same approach, complexes of carboxyl-DTF **7** with Zn(II) ions were also produced as a yellow powder.

**Figure 1:**
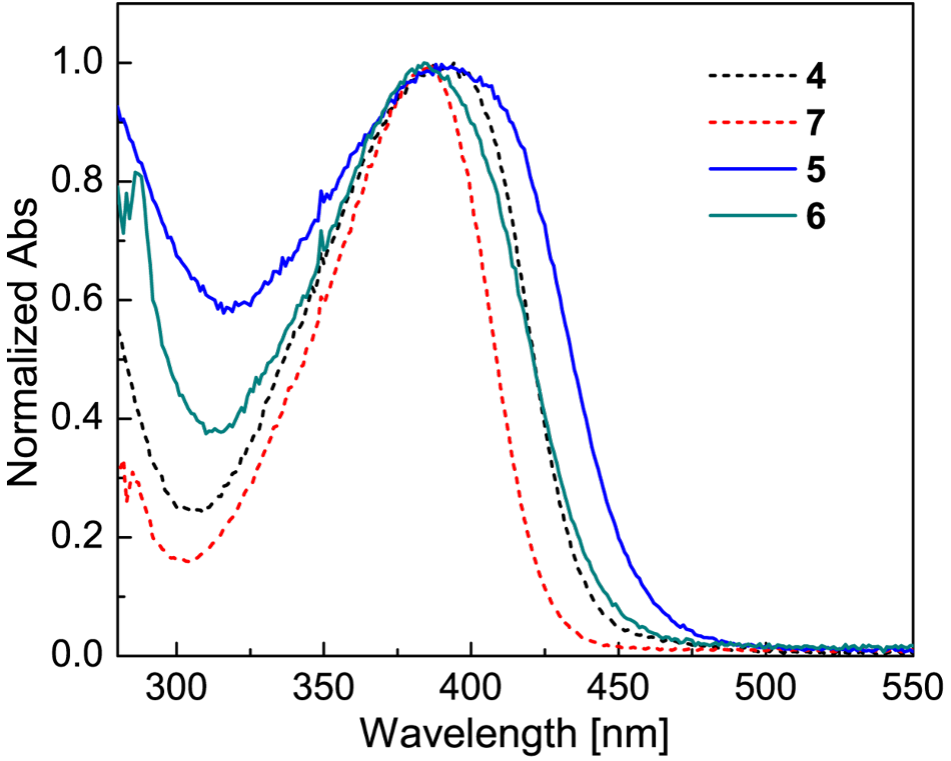
UV–vis spectra of TTFVs **5** and **6** (solid lines) and DTF **4** and **7** (dashed lines). Compounds **4** and **5** were measured in CH_2_Cl_2_, while compounds **6** and **7** were in THF.

The electronic properties of TTFVs **5** and **6** as well as their DTF precursors **4** and **7** were investigated by UV–vis absorption spectroscopy. Figure 1 shows the UV–vis absorption spectra of these compounds, in which the maximum absorption wavelengths (λ_max_) of all the compounds appear to be nearly identical at ca. 385 nm. There are, however, slight variations in the cut-off energies of long-wavelength absorption bands. The origins of these long-wavelength absorption bands are mainly due to HOMO to LUMO, HOMO to LUMO+1, and HOMO−1 to LUMO+1 transitions according to time-dependent density functional theory (TD-DFT) calculations (see the Supporting Information File 1 for details). The UV–vis data indicates that the degrees of π-delocalization for the TTFVs and DTF compounds are quite similar. This result is congruous with the fact that diphenyl-TTFVs generally prefer a twisted *cisoid* conformation in the ground state [12,15–16], which in theory significantly disrupts the π-delocalization within the molecules. Therefore, even though the molecular sizes of TTFVs **5** and **6** are doubled in comparison with their DTF precursors **4** and **7**, the degrees of π-electron delocalization in these molecules are still retained at a similar level in the ground state.

**Figure 2:**
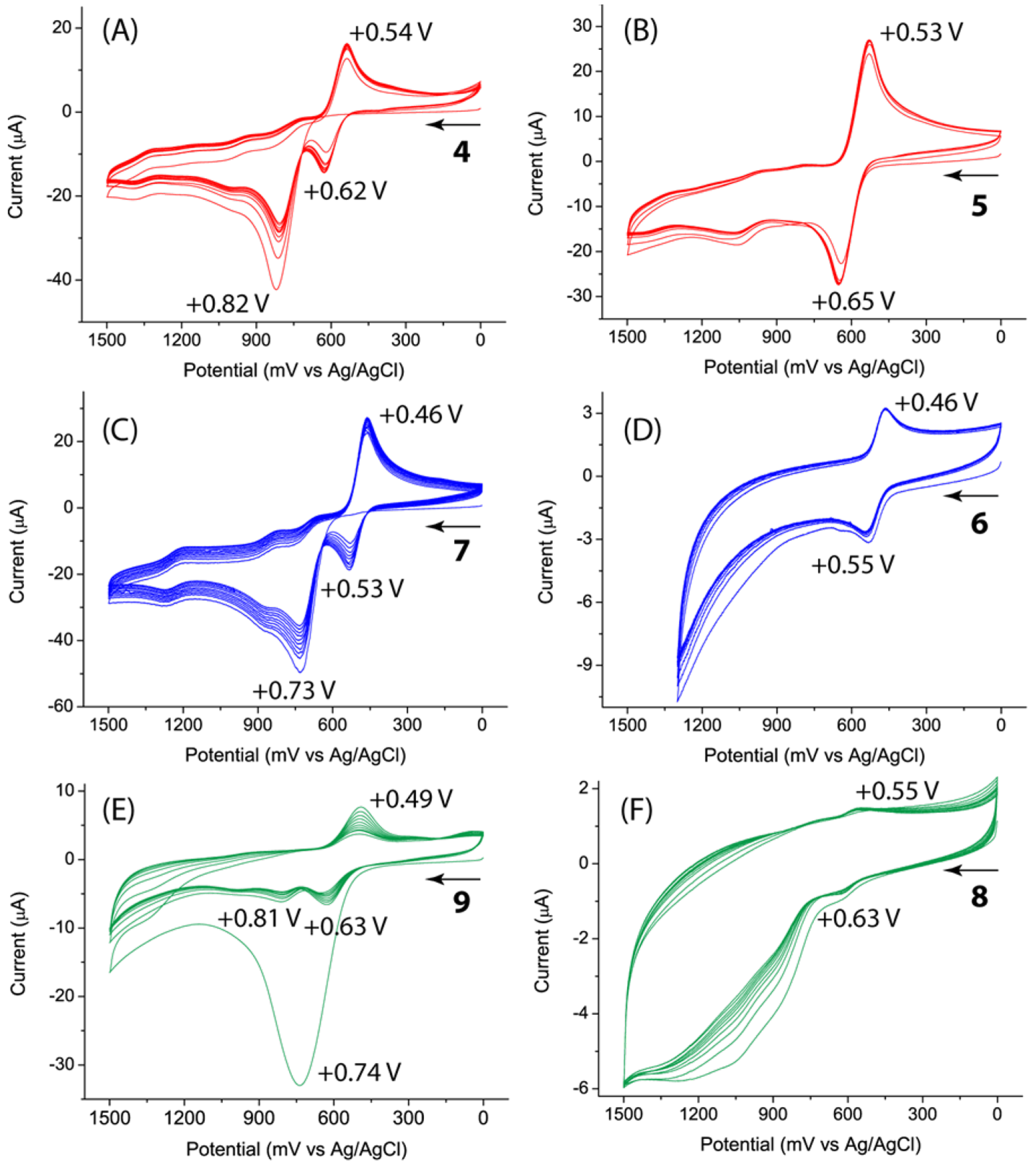
Cyclic voltammograms of compounds **4**–**9**. Experimental conditions: supporting electrolyte: Bu_4_NBF_4_ (0.1 M), working electrode: glassy carbon, counter electrode: Pt wire, reference electrode: Ag/AgCl (3 M NaCl), scan rate: 200 mV s^−1^. Compounds **4** (1.5 mM), **5** (0.73 mM), **8**, and **9** were measured in CH_2_Cl_2_. Compounds **6** (0.76 mM) and **7** (1.5 mM) were measured in CH_3_CN.

The electrochemical redox properties of compounds **4**–**9** were characterized by cyclic voltammetry, and the detailed cyclic voltammograms are shown in Figure 2. For methyl ester-appended DTF **4** (Figure 2A) an anodic peak was observed at +0.82 V in the first cycle of scan, which is due to the single-electron oxidation of the dithiole moiety into the dithiolium radical cation [15–16]. In the reverse scan, a cathodic peak emerged at +0.54 V which is assigned to the bielectronic reduction of the TTFV product electrochemically generated on the electrode surface via the DTF dimerization reaction [15–16]. In the following scan cycles, the redox wave pair characteristic of TTFV at *E*_pa_ = +0.62 V and *E*_pc_ = +0.54 V was found to gradually increase in intensity as a result of increasing electrochemical dimerization. The same electrochemical patterns can be seen in the cyclic voltammograms of carboxyl-DTF **7** and Zn-DTF complex **9** (Figure 2C and 2E); however, their redox potentials showed a slight degree of variation. Experimentally, the cyclic voltammogram of **9** was determined from its solid thin film compressed on the working electrode surface. It is interesting to note that Zn-DTF complex **9** retained the redox activity and electrochemical reactivity of DTF even in the solid state. The cyclic voltammograms of compounds **5** and **6** both featured a reversible redox wave pair due to the simultaneous bielectronic transfers occurring at the TTFV moieties (Figure 2B and 2D). In the cyclic voltammogram of Zn-TTFV coordination polymer **8** (measured from a solid film prepared in the same way as **9**), the redox wave pair of TTFV is discernible but much weaker than that of Zn-DTF **9** (Figure 2F), suggesting that the electrochemical activity of the coordination polymer is considerably reduced in comparison with the smaller-sized Zn-DTF complex.

**Figure 3:**
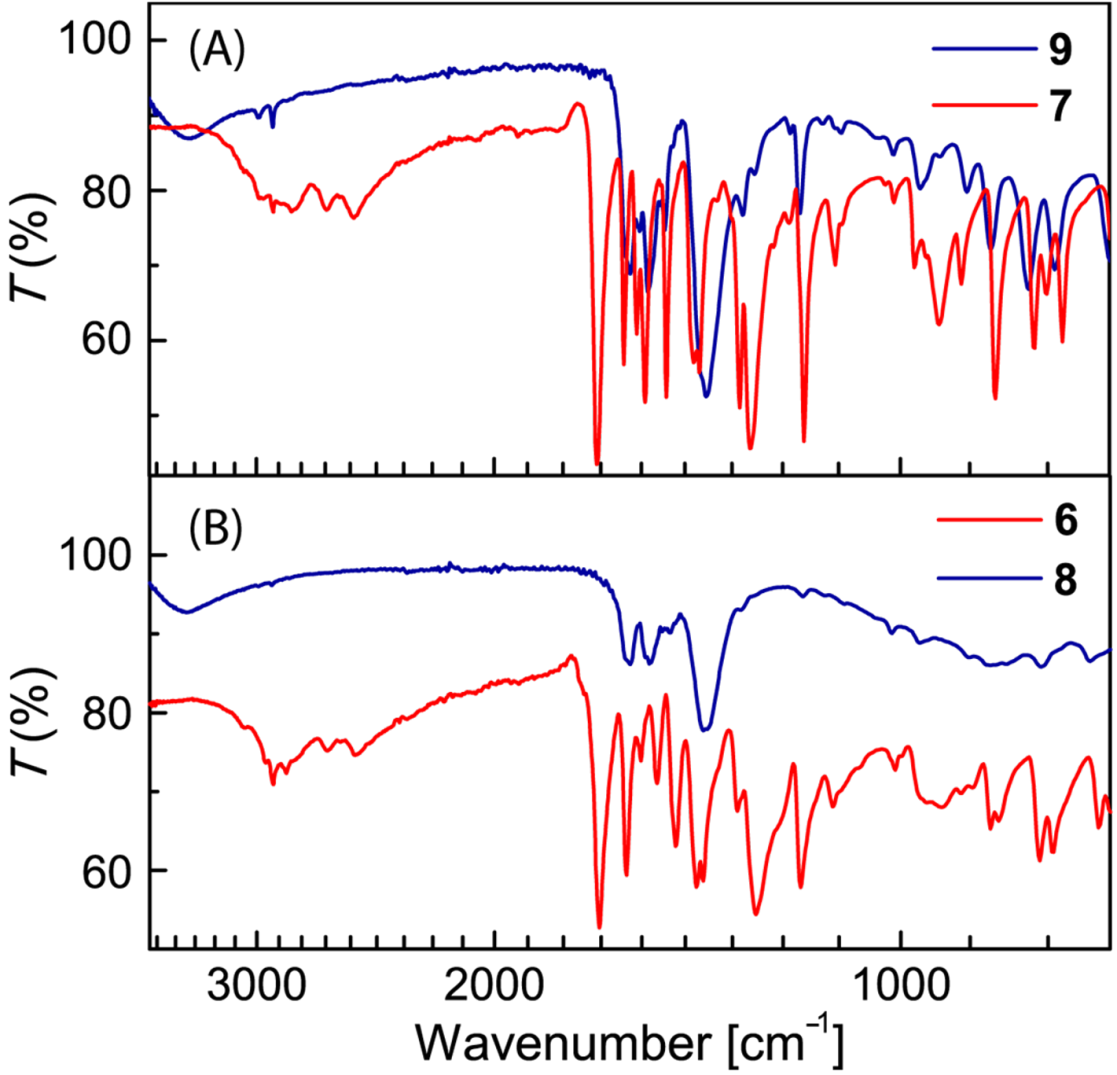
FTIR spectra of compounds **6–9**.

**Figure 4:**
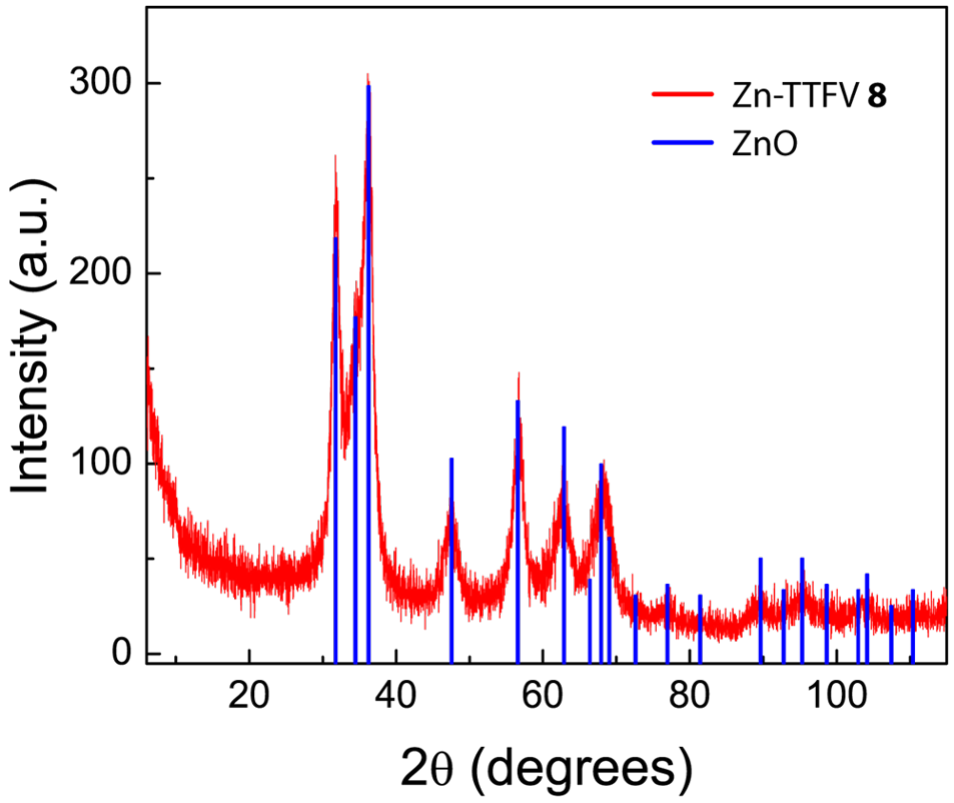
PXRD patterns of the Zn-TTFV coordination polymer **8** (red line) in comparison with the diffraction data of zincite ZnO (blue bars).

The structural properties of Zn-TTFV coordination polymer **8** and Zn-DTF complex **9** were examined by IR spectroscopy (Figure 3). Compared with the IR spectra of carboxyl-TTFV **6** and carboxyl-DTF **7**, the vibrational bands of free carboxyl groups were clearly absent in the spectra of **8** and **9**, confirming that the carboxyl groups were completely coordinated with Zn(II) ions. The crystalline properties of coordination polymer **8** were examined by powder X-ray diffraction (PXRD) analysis. The diffraction patterns shown in Figure 4 confirm that coordinate polymer **8** possesses crystallinity in the solid state. Actually, the diffraction patterns were found to bear resemblance to those of zincite ZnO. Such crystalline features hence point to a possibility of the coordination polymer to take some kind of framework-like structures in the solid state with certain microscopic porosity.

**Figure 5:**
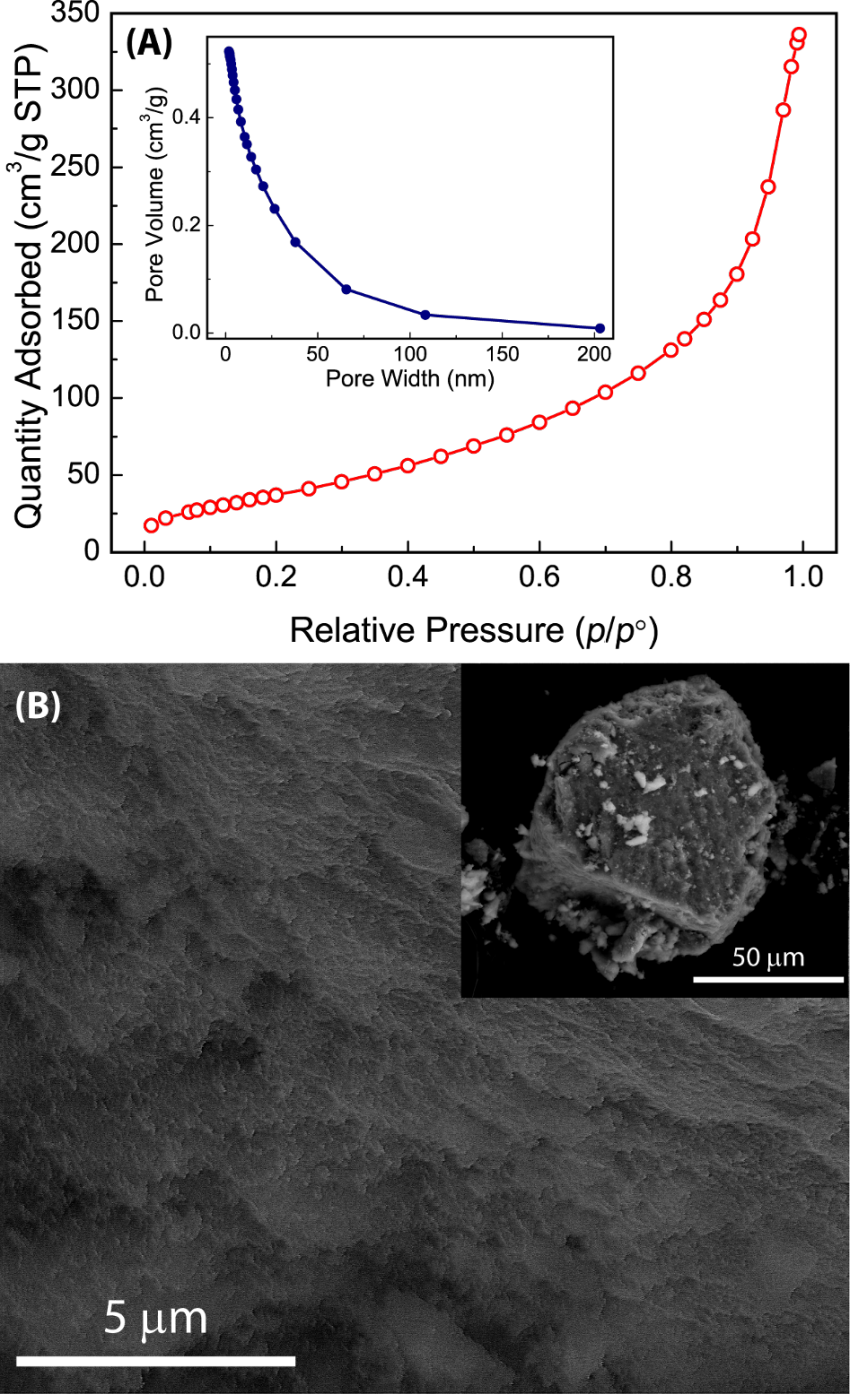
(A) Nitrogen adsorption isotherm of coordination polymer **8** measured at 77 K. Inset: pore size distributions. (B) SEM image of the powder of **8**.

To assess the porous properties of Zn-TTFV **8**, nitrogen gas adsorption analysis was conducted at 77 K. The adsorption isotherm shown in Figure 5A indicates a Type-II adsorption behaviour. Application of the Brunauer–Emmett–Teller (BET) model gave a BET surface area of 148.2 m^2^ g^−1^ and an adsorption average pore diameter of 10.2 nm. The pore size distribution analysis data revealed that Zn-TTFV coordination polymer **8** carried microporosity primarily in the range of tens of nanometers. Scanning electron microscopic (SEM) imaging was performed on the particles of **8** to show some kind of crystalline-like micromorphology. In line with the gas adsorption results, there were no relatively large pores on the micron scale observable in the particles (see the inset of Figure 5B). On the submicron scale, however, corrugated microporous features could be clearly observed (see Figure 5B). To understand the origin of the micropores, nitrogen gas adsorption experiments were performed on Zn-DTF complex **9**. The experimental results did not lead to any meaningful measurements of BET surface area and microporosity, indicating a lack of porous structures in the solid of **9**. The major structural difference between Zn-DTF **9** and Zn-TTFV **8** is that **8** assumes crystalline polymeric frameworks as evidenced by PXRD analysis, whereas **9** is in the form of small clusters and amorphous (see Figure S10 in Supporting Information File 1 for the detailed PXRD data of **9**). It is therefore reasonable to propose that the microporosity in **8** is directly related to the coordination polymer structure.

**Figure 6:**
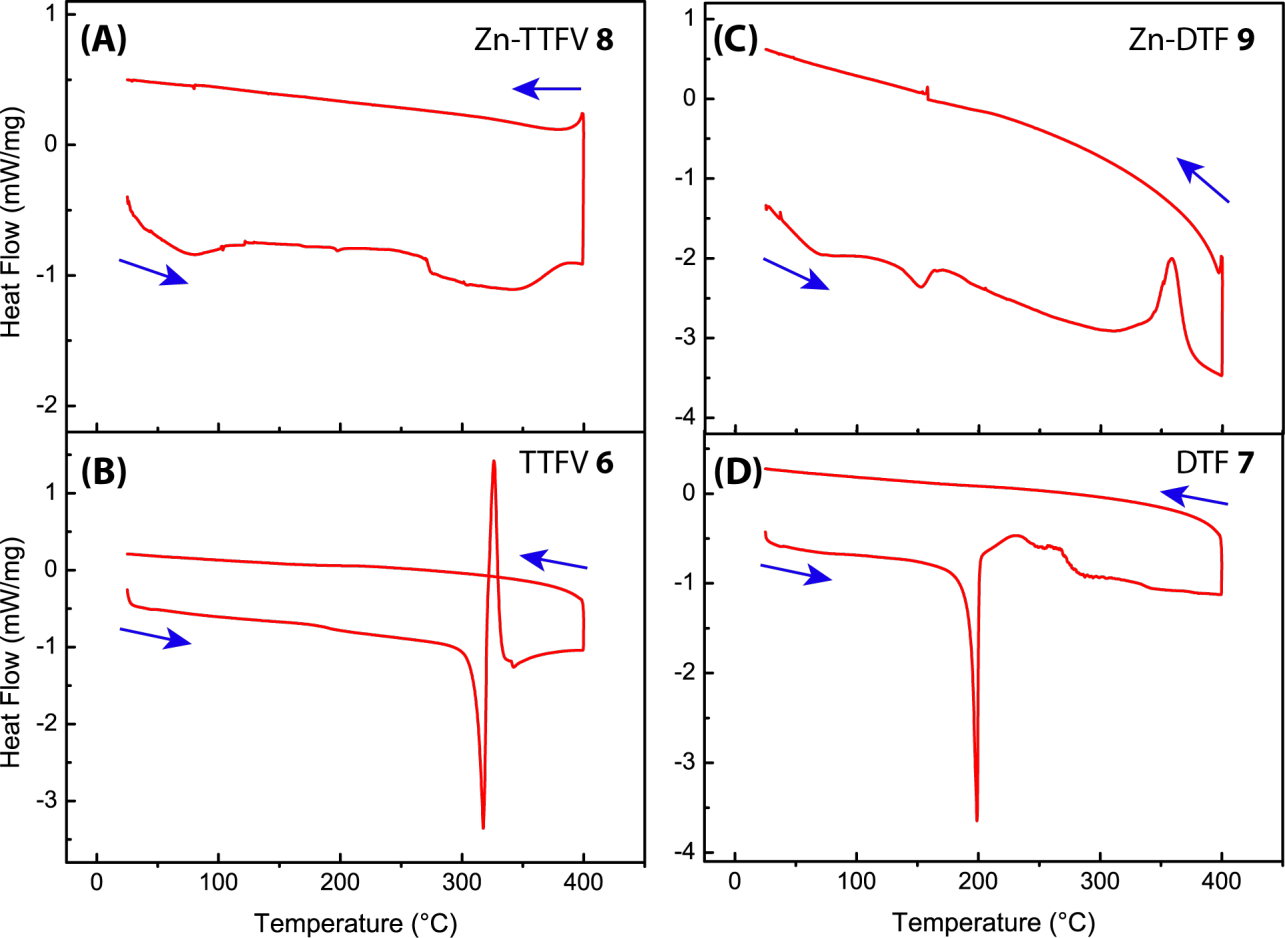
DSC traces of compounds **6**–**9** measured under a nitrogen atmosphere. Scan range: 25–400 °C, scan rate: 10 °C min^−1^.

Finally, the thermal stability of coordination polymer **8** and Zn-DTF **9** were evaluated by differential scanning calorimetric (DSC) analysis, and detailed DSC traces are illustrated in Figure 6. The DSC data of Zn-TTFV **8** (Figure 6A) manifested very good thermal stability up to 400 °C, without any significant melting or decomposition except a slight phase transition at 272 °C. Comparatively, the DSC trace of carboxyl-TTFV ligand **6** showed a distinctive melting process at 317 °C, which was immediately followed by a prominent sharp exothermic peak at 326 °C (Figure 6B). The exothermic process is possibly due to a chemical reaction(s); however, the exact reactivity awaits further investigation to clearly elucidate. Zn-DTF **9** gave a moderate endothermic peak at 152 °C and a significant exothermic peak at 358 °C (Figure 6C). For carboxyl-DTF **7**, a notable melting point was observed at 199 °C, and the melting was followed by certain exothermic processes in the range of 200 to 285 °C. The DSC results indicated that the formation of Zn-TTFV coordinate polymer could give rise to considerably improved thermal stability, a property particularly beneficial for practical device and material applications.

## Conclusion

In summary, we have synthesized carboxylated diphenyl-TTFV **6** and phenyl-DTF **7** as redox-active ligands to complex with Zn(II) ions. The electronic and electrochemical properties of the TTFV and DTF compounds were found to be in line with other related TTFV and DTF derivatives. Of great interest is that the complexes with Zn(II) ions retain the redox activity and electrochemical reactivity of their TTFV and DTF ligands in the solid state. Another significant added value is the thermal robustness of the Zn-TTFV coordination polymer. Collectively, the good electrochemical and thermal properties point to a promising prospect for them to be further developed into practically useful organic–inorganic hybrid materials through the coordination polymer approach. The Zn-TTFV coordination polymer was also found to be crystalline in nature. At this stage, meaningful single-crystal diffraction data has not yet been successfully determined. Without such data clear understanding of the detailed solid-state structural properties cannot be established. Our future work is moving towards tuning the side groups of the TTFV ligand to produce Zn complexes with better crystallinity. Finally, the Zn-TTFV coordination polymer exhibited significant microporosity and surface area. Overall, our current studies have cast a light on the fundamental redox and solid-state properties of the class of TTFV-based organic–inorganic hybrid materials, and the findings disclosed in this article should offer useful guidance to further material design and development.

## Experimental

Chemicals were purchased from commercial suppliers and used directly without purification. All reactions were conducted in standard, dry glassware and under an inert atmosphere of nitrogen unless otherwise noted. Evaporation and concentration were carried out with a water-aspirator. Flash column chromatography was performed with silica gel 60 (240-400 mesh). Thin-layer chromatography (TLC) was carried out with silica gel F254 covered on plastic sheets and visualized by UV light. Melting points were measured on a SRS OptiMelt melting point apparatus. ^1^H and ^13^C NMR spectra were measured on a Bruker Avance III 300 MHz multinuclear spectrometer. Chemical shifts (δ) are reported in ppm downfield relative to the signal of the internal reference SiMe_4_. Coupling constants (*J*) are given in Hz. Infrared spectra (IR) were recorded on a Bruker Alfa spectrometer. HRMS analyses were performed on an Agilent 6230 TOF LC/MS instrument using an APPI ionizer. UV–vis absorption spectra were measured on a Cary 6000i spectrophotometer. Cyclic voltammetric analyses were carried out in a standard three-electrode setup controlled by a BASi epsilon workstation. Differential scanning calorimetric (DSC) analyses were performed on a Mettler-Toledo DSC1 calorimeter. Powder X-ray diffraction (PXRD) data was collected on a Rigaku Ultima IV diffractometer equipped with a copper X-ray source with a wavelength of 1.54 nm. Scanning electron microscopy (SEM) was performed on an FEI MLA 650 FEG microscope. BET surface area and pore size analyses were performed on a Micromeritics TriStar II Plus instrument. The degassing was done on a Flow Prep 060 instrument. The calculations were carried out with the MicroActive for TriStar II Plus software (Version 2.02). Thione **2** was prepared according to the procedures we reported previously [22–23].

**DTF 4:** A mixture of methyl 4-formylbenzoate (**3**, 1.83 g, 11.1 mmol) and thione **2** (3.03 g, 13.4 mmol) in P(OMe)_3_ (100 mL) was stirred and heated at 105 °C for 3 h. The excess P(OMe)_3_ was removed by vacuum distillation. The residue was purified by silica column chromatography (EtOAc/hexanes, 1:9) to afford compound DTF **4** (2.93 g, 8.55 mmol, 77%) as a yellow crystalline solid. mp 88.6–90.9 °C; ^1^H NMR (300 MHz, CDCl_3_) δ 8.01 (d, *J* = 8.4 Hz, 2H), 7.25 (d, *J* = 8.3 Hz, 2H), 6.51 (s, 1H), 3.91 (s, 3H), 2.44 (d, *J* = 2.8 Hz, 6H) ppm; ^13^C NMR (75 MHz, CDCl_3_) δ 166.8, 140.5, 136.2, 129.9, 127.9, 126.7, 126.3, 124.5, 113.3, 52.0, 19.0, 18.9 ppm; FTIR (neat): 2914, 1704, 1599, 1567, 1545, 1492, 1422, 1265, 1175, 1096, 851, 798, 693, 470 cm^−1^; APPI–HRMS (*m/z*, positive mode): [M^+^] calcd for C_14_H_14_O_2_S_4_, 341.9877; found, 341.9878.

**TTFV 5:** A mixture of DTF **4** (0.25 g, 0.73 mmol) and I_2_ (0.55 g, 2.2 mmol) in CH_2_Cl_2_ (100 mL) was stirred at rt overnight. Then a satd Na_2_S_2_O_3_ solution (aq, 90 mL) was added. The mixture was stirred for another 3 h at rt. The organic layer was separated, washed with H_2_O, dried over MgSO_4_, and concentrated under vacuum. The residue was purified by silica column chromatography (EtOAc/hexanes, 1:4) to afford compound **5** (0.20 g, 0.29 mmol, 80%) as a yellow solid. mp 183.9–185.4 °C; ^1^H NMR (300 MHz, CDCl_3_) δ 7.97 (d, *J* = 8.7 Hz, 4H), 7.46 (d, *J* = 8.7Hz, 4H), 3.89 (s, 6H), 2.44 (s, 6H), 2.38 (s, 6H) ppm; ^13^C NMR (75 MHz, CDCl_3_) δ 166.6, 141.1, 140.3, 130.1, 129.1, 127.9, 126.1, 125.5, 122.9, 52.0, 18.9, 18.8 ppm; FTIR (neat): 2942, 2918, 1709, 1600, 1519, 1473, 1430, 1273, 1182, 1107, 766, 713, 465 cm^−1^; APPI–HRMS (*m/z*, positive mode): [M^+^] calcd for C_28_H_26_O_4_S_8_, 681.9597; found, 681.9584.

**Carboxylated TTFV 6:** A mixture of TTFV **5** (50.0 mg, 0.0732 mmol) and NaOH (46.9 mg, 1.17 mmol) in MeOH/H_2_O (40 mL, 3:1) was stirred at 75 °C overnight. The solvent MeOH was removed under vacuum, and the residue was diluted to 50 mL with H_2_O and acidified to pH 4 with HCl (aq). The precipitate formed was extracted with EtOAc, washed with H_2_O, dried over MgSO_4_, and concentrated under vacuum to afford compound **6** (40.3 mg, 0.0615 mmol, 84%) as a yellow solid. mp 292.9–295.7 °C; ^1^H NMR (300 MHz, DMSO-*d*_6_) δ 7.94 (d, *J* = 8.5 Hz, 4H), 7.48 (d, *J* = 8.4 Hz, 4H), 2.48 (s, 6H), 2.40 (s, 6H) ppm; ^13^C NMR (75 MHz, DMSO-*d*_6_) δ 166.7, 140.0, 139.1, 130.0, 128.8, 126.8, 125.9, 125.1, 122.7, 18.2, 18.2 ppm; FTIR (neat): 2916-2536 (br), 1672, 1596, 1515, 1467, 1416, 1280, 1186, 789, 541, 469 cm^−1^; APPI–HRMS (*m/z*, negative mode) [M^−^] calcd for C_26_H_22_O_4_S_8_, 653.9284; found, 653.9293.

**Carboxylated DTF 7:** A mixture of DTF **4** (0.30 g, 0.88 mmol) and NaOH (0.56 g, 14 mmol) in MeOH/H_2_O (240 mL, 3:1) was stirred at 75 °C overnight. The solvent MeOH was removed under vacuum, and the residue was diluted to 100 mL with H_2_O and acidified to pH 4 with HCl (aq). The precipitate formed was subjected to suction filtration to afford compound **7** (0.24 g, 0.73 mmol, 84%) as a yellow solid. mp 191.0–193.2 °C; ^1^H NMR (300 MHz, DMSO-*d*_6_) δ 12.86 (s, 1H), 7.94 (d, *J* = 8.4 Hz, 2H), 7.32 (d, *J* = 8.3 Hz, 2H), 6.82 (s, 1H), 2.47 (s, 3H), 2.45 (s, 3H) ppm; ^13^C NMR (75 MHz, DMSO-*d*_6_) δ 166.9, 139.7, 134.6, 129.7, 127.4, 127.3, 126.3, 122.7, 113.7, 18.3, 18.1 ppm; FTIR (neat): 2914–2540 (br), 1677, 1602, 1567, 1545, 1490, 1408, 1291, 1178, 850, 796, 505, 470 cm^−1^; APPI−HRMS (*m/z*, negative mode) [M^−^] calcd for C_13_H_12_O_2_S_4_, 327.9720; found, 327.9727.

**Zn-TTFV 8:** A solution of TTFV **6** (0.12 g, 0.18 mmol) and Zn(NO_3_)_2_∙6H_2_O (0.11 g, 0.37 mmol) in EtOH (350 mL) was added into a beaker, which was placed in a larger beaker containing Et_3_N/EtOH (40 mL, 1:1). The larger beaker was sealed and left standing for 4 days. The precipitate formed within the smaller beaker was collected by centrifugation and rinsed with EtOH to afford Zn-TTFV **8** (50.8 mg) as a yellow solid. FTIR (neat): 3381, 1586, 1535, 1400, 857, 787 cm^−1^.

**Zn-DTF 9:** A solution of DTF **7** (20.0 mg, 0.0610 mmol) and Zn(NO_3_)_2_∙6H_2_O (21.8 mg, 0.0733 mmol) in EtOH (40 mL) was added into a vial, which was placed in a jar containing Et_3_N/EtOH (6 mL, 1:2). The jar was sealed and left standing for 2 days. The precipitate formed in the vial was collected by centrifugation and rinsed with EtOH to afford Zn-DTF **9** (15.1 mg) as a yellow solid. FTIR (neat): 3360, 2990, 2916, 1585, 1559, 1538, 1494, 1392, 1187, 803, 768, 472 cm^−1^.

## Supporting Information

File 1 ^1^H and ^13^C NMR spectra of compounds **4**–**7**, PXRD data of **8** and **9**, thermal gravimetric analysis (TGA) data of **8**, and time-dependent (TD) DFT calculation results for compounds **6** and **7**.
