# Supplementary material for: Carboxylated dithiafulvenes and tetrathiafulvalene vinylogues: synthesis, electronic properties, and complexation with zinc ions
Source: Beilstein J Org Chem. 2015 Jun 3;11:957–65. doi: 10.3762/bjoc.11.107 (PMC4464393; doi:10.3762/bjoc.11.107)
Supplement: File 1 — 1H and 13C NMR spectra of compounds 4–7, PXRD data of 8 and 9, thermal gravimetric analysis (TGA) data of 8, and time-dependent (TD) DFT calculation results for compounds 6 and 7. [file Beilstein_J_Org_Chem-11-957-s001.pdf]

Supporting Information

for

Carboxylated dithiafulvenes and tetrathiafulvalene vinylogues: synthesis, electronic properties, and complexation with zinc ions

Yunfei Wang and Yuming Zhao*

Address: Department of Chemistry, Memorial University, St. John's, A1B 3X7, NL, Canada

Email: Yuming Zhao* - yuming@mun.ca

*Corresponding author

^{1}H and ^{13}C NMR spectra of compounds 4–7, PXRD data of 8 and 9, thermal gravimetric analysis (TGA) data of 8, and time-dependent (TD) DFT calculation results for compounds 6 and 7.

Table of contents

1. **NMR spectra of compounds 4–7** S2

2. **PXRD data for Zn-TTFV 8 and Zn-DTF 9** S10

3. **TGA data for Zn-TTFV 8** S11

4. **Results of density functional theory calculations** S12

1. NMR Spectra of compounds 4–7

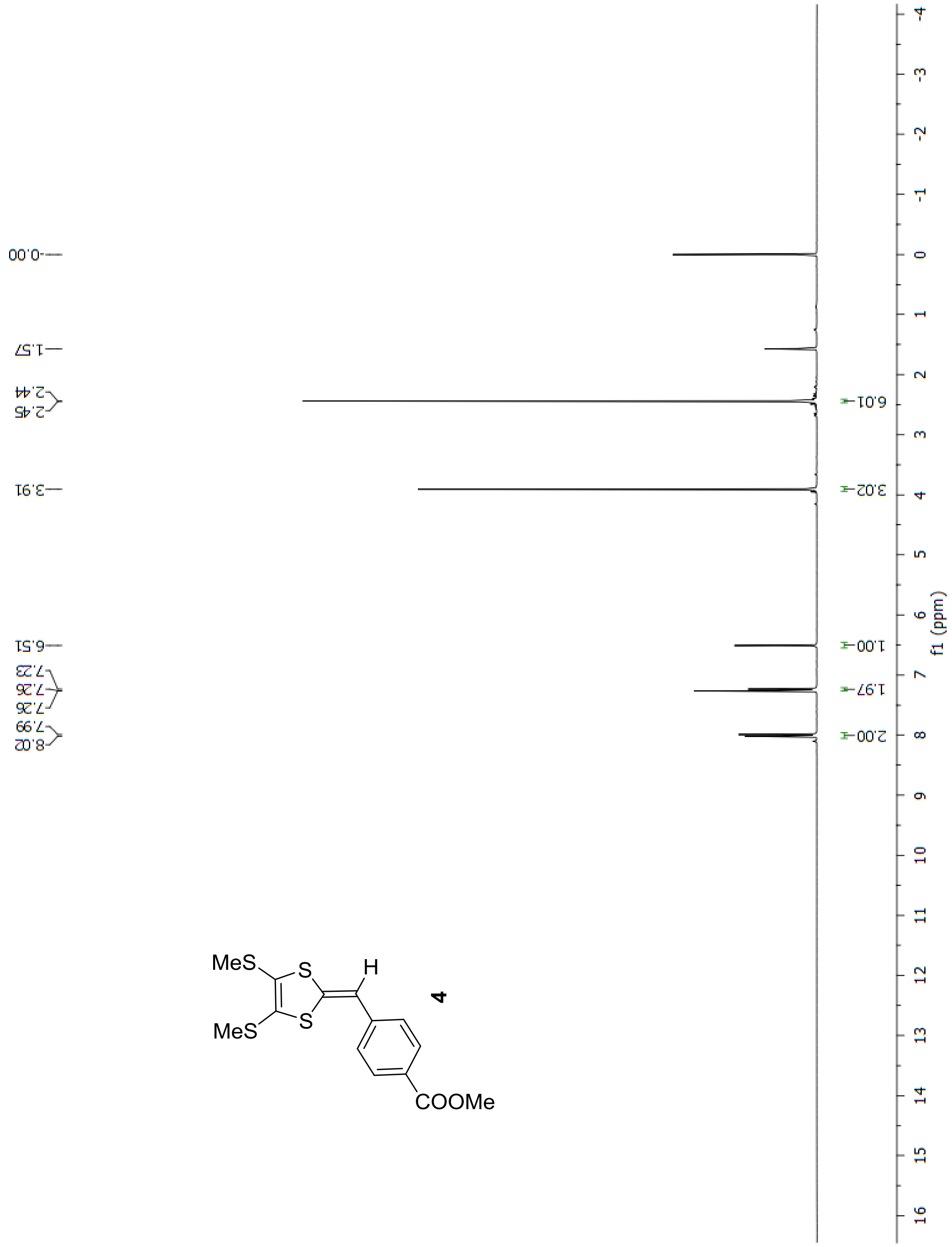

Figure S1: ^{1}H NMR (300 MHz, $CDCl_3$) spectrum of compound **4**.

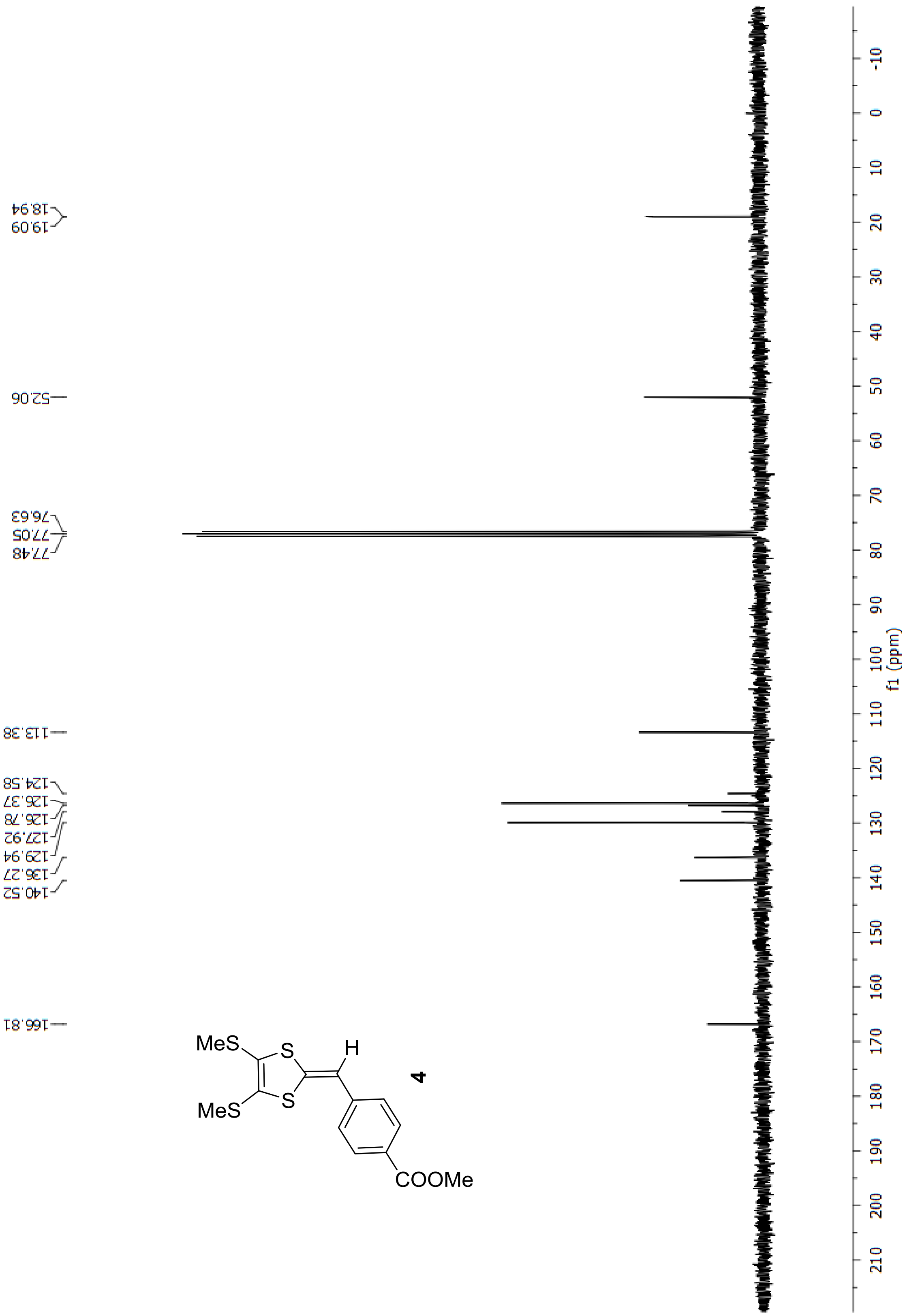

Figure S2: ^{13}C NMR (75 MHz, $CDCl_3$) spectrum of compound **4**.

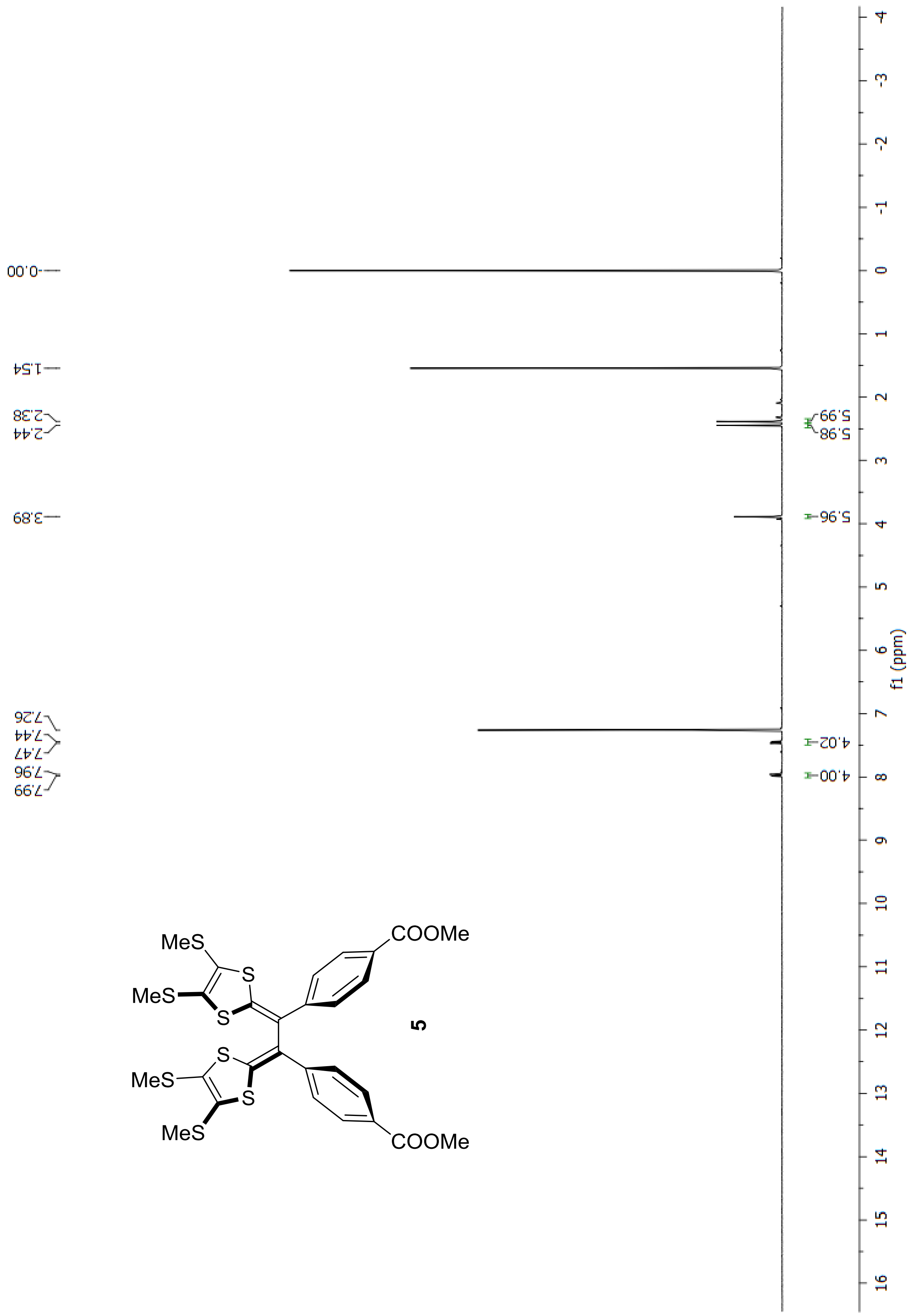

Figure S3: ^{1}H NMR (300 MHz, $CDCl_3$) spectrum of compound **5**.

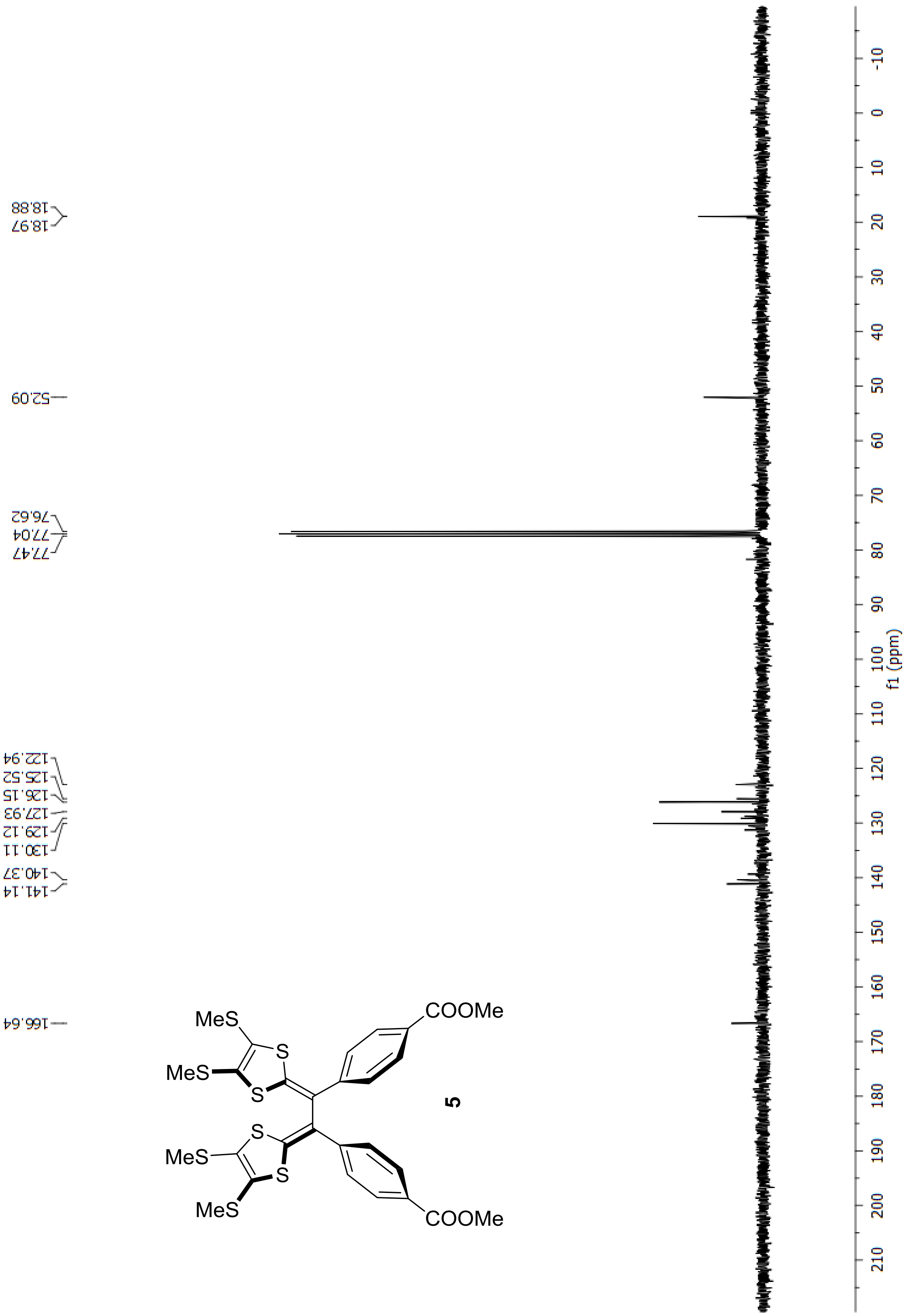

Figure S4: ^{13}C NMR (75 MHz, $CDCl_3$) spectrum of compound **5**.

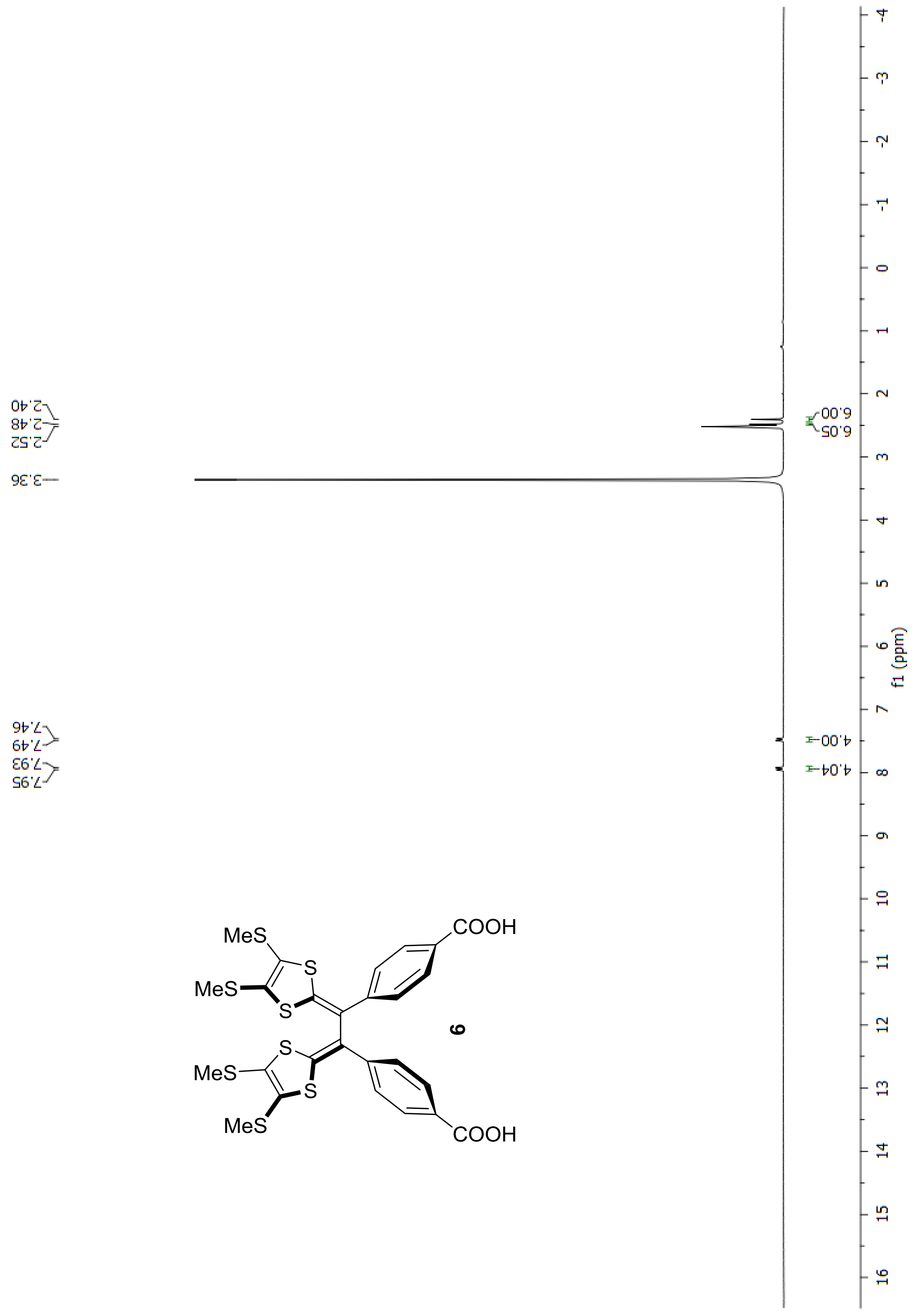

Figure S5: ^{1}H NMR (300 MHz, DMSO-d_6) spectrum of compound **6**.

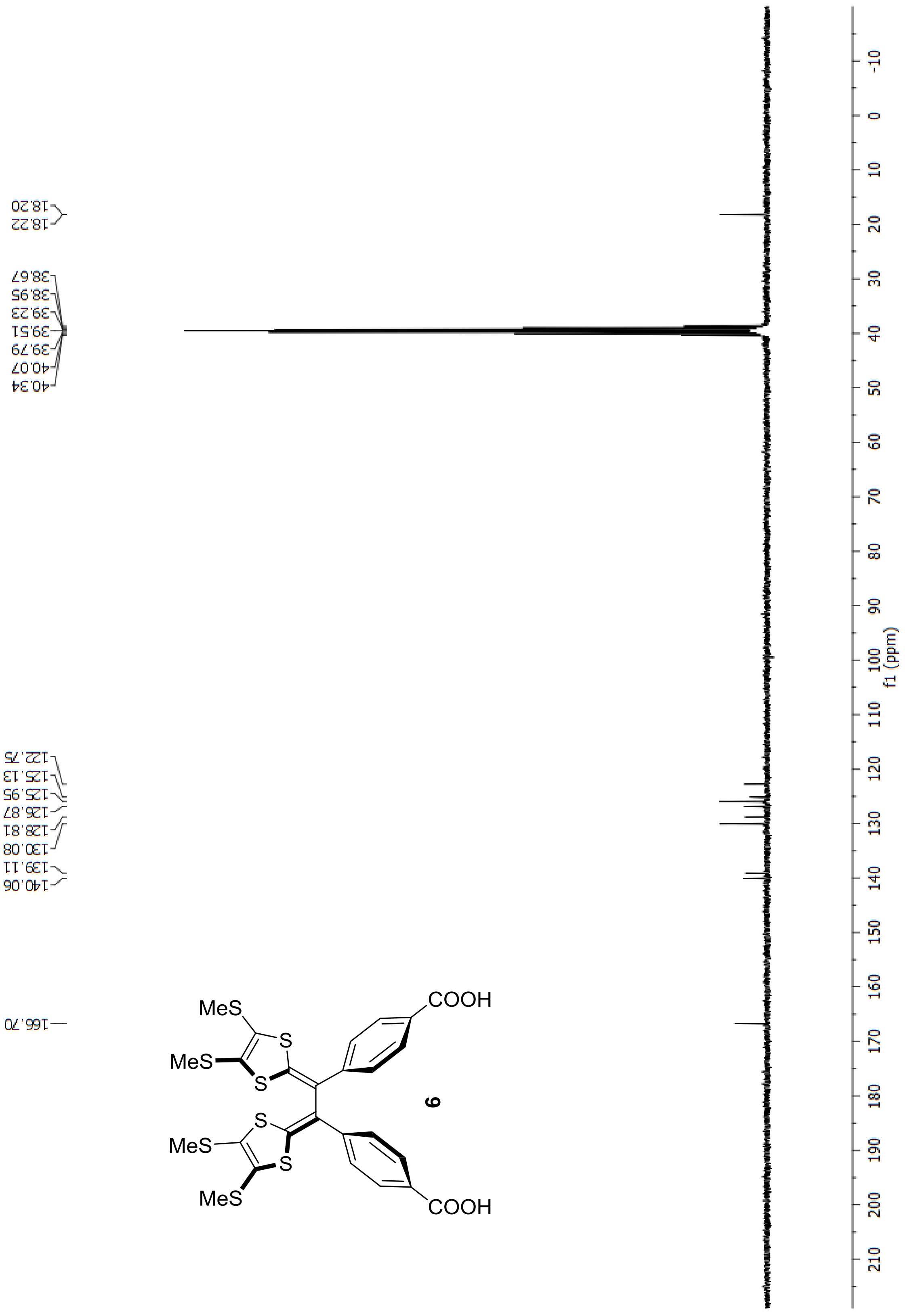

Figure S6: ^{13}C NMR (75 MHz, DMSO-d_6) spectrum of compound **6**.

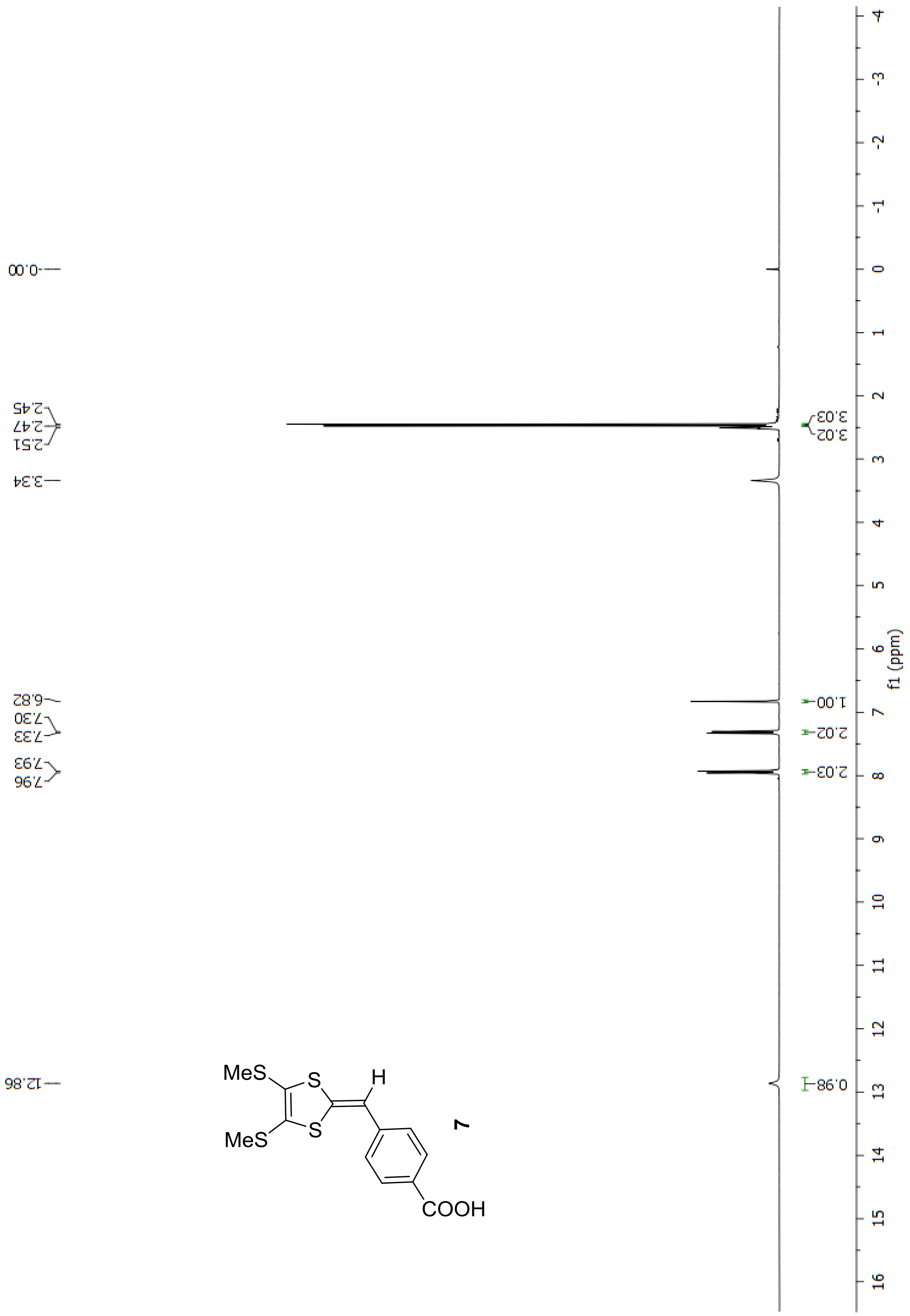

Figure S7: ^{1}H NMR (300 MHz, DMSO-d_6) spectrum of compound **7**.

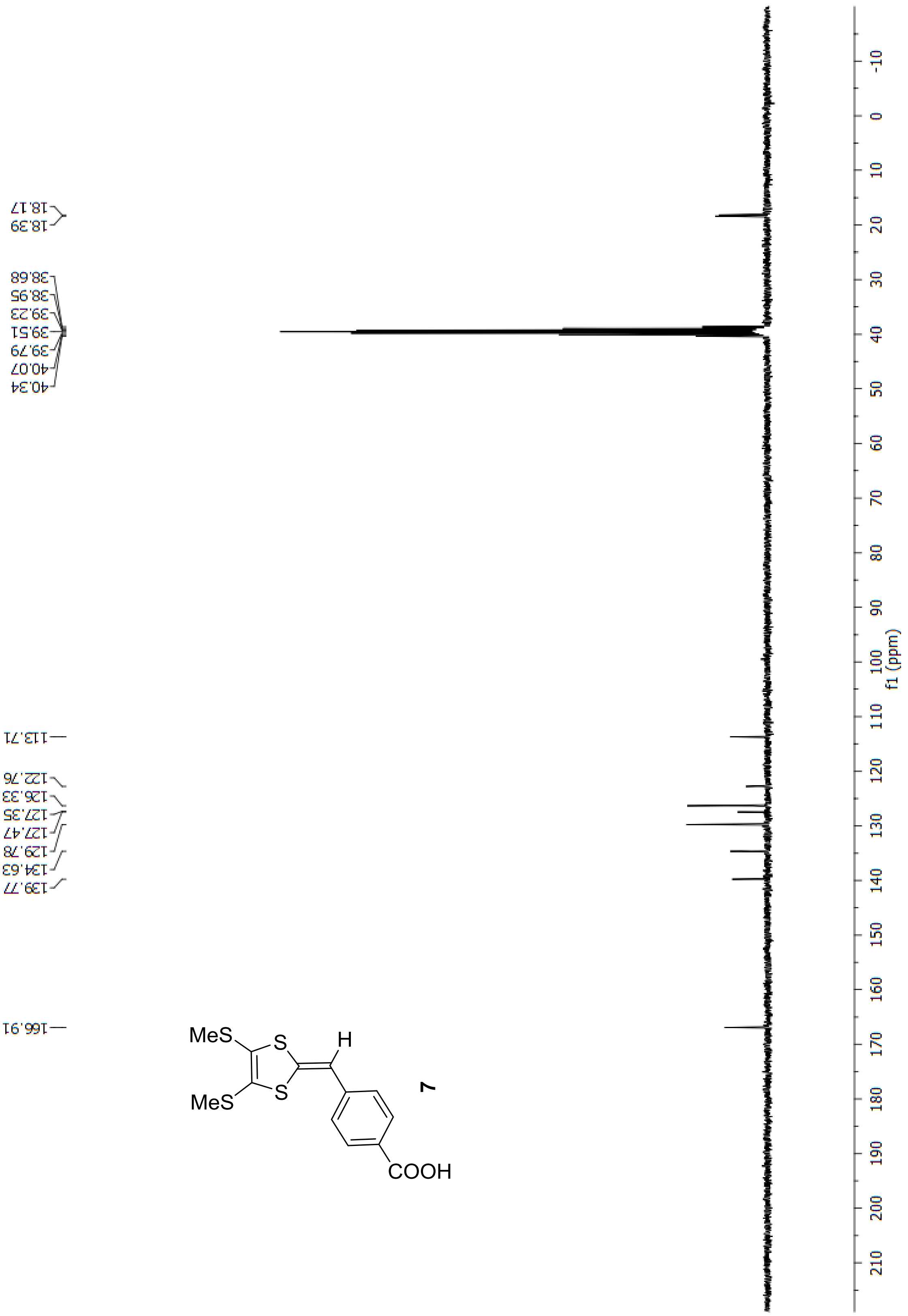

Figure S8: ^{13}C NMR (75 MHz, DMSO-d_6) spectrum of compound **7**.

2. PXRD data for Zn-TTFV 8 and Zn-DTF 9

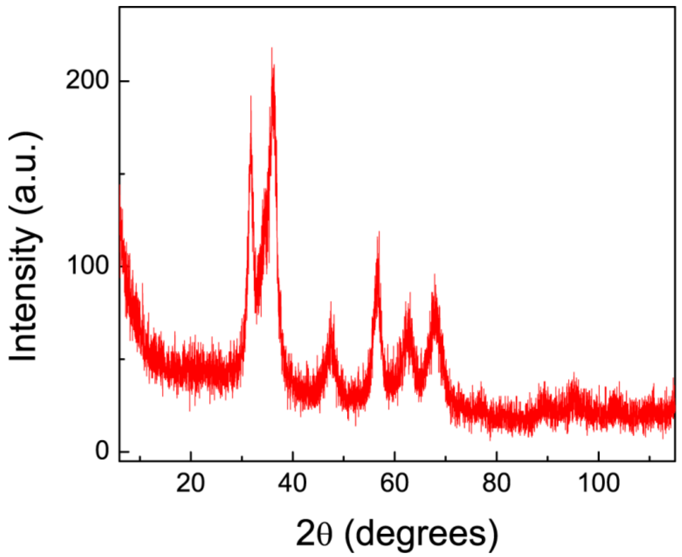

Figure S9: PXRD patterns of the Zn-TTFV coordination polymer **8** after oxidation with iodine vapour for 30 min.

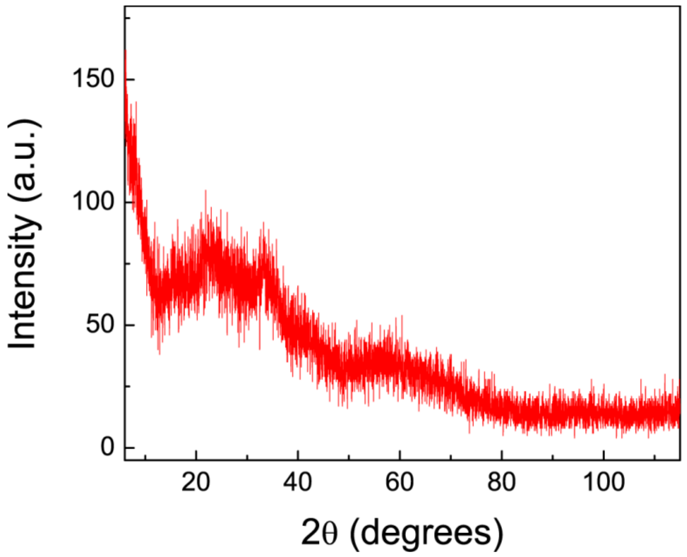

Figure S10: PXRD patterns of the Zn-DTF complex **9**.

3. TGA data for Zn-TTFV 8

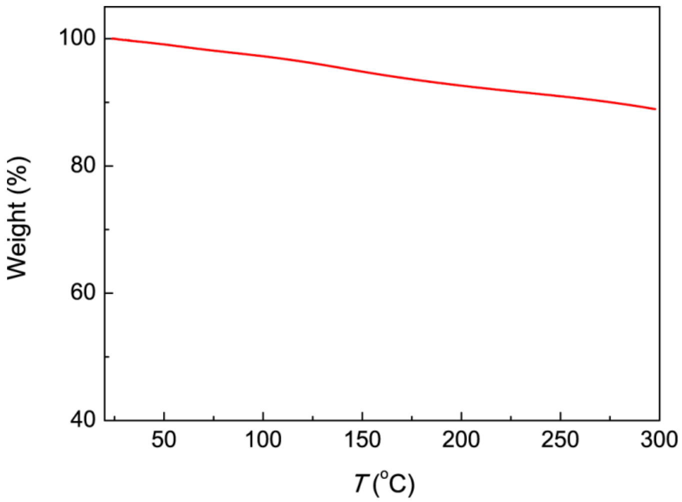

Figure S11: TGA profile of Zn-TTFV-Zn coordination polymer **8** measured under N_2. Scan rate: 5 °C min^{-1}.

4. Results of density functional theory calculations

Density functional theory (DFT) calculations were performed on compounds **6** and **7** using the Gaussian 09 software package (Gaussian Inc.). Geometry optimizations were carried out at the B3LYP/6-31G(d) level of theory at first and the obtained structures were validated to be the global energy minima by frequency check (i.e., zero imaginary frequency). The optimized structures were then subjected to TD-DFT calculations at the same level of theory, taking only singlet transitions into consideration (nstate = 20).

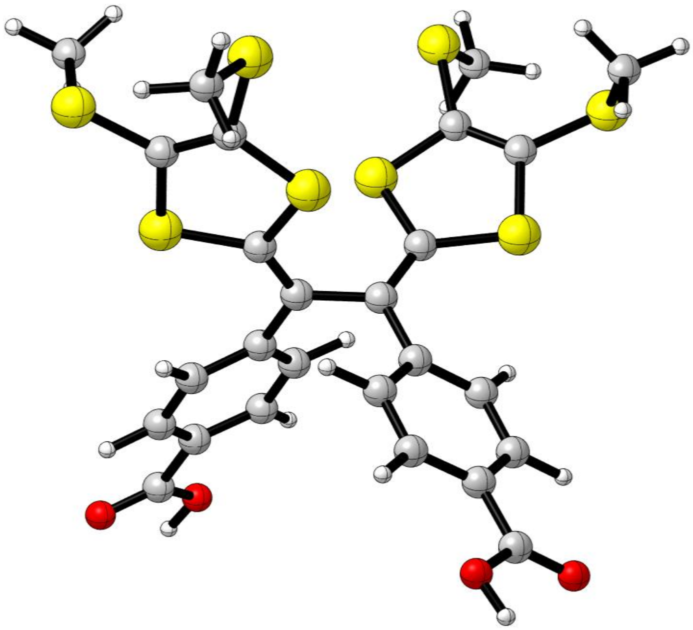

Figure S12: Optimized geometry of compound **6** at the B3LYP/6-31G(d) level.

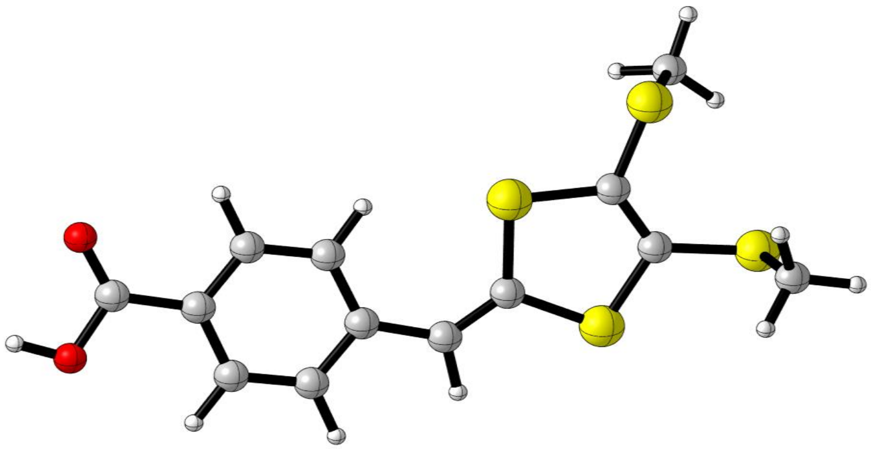

Figure S13: Optimized geometry of compound **7** at the B3LYP/6-31G(d) level.

Table S1: Summary of TD-DFT calculation results for compounds **6** and **7***.

Entry	λ_{cacld} (nm)	*f*	MO Character	λ_{obs} (nm)
**6**	412.24	0.1565	H-1 → L+1 (0.15720), H → L (0.68251)	386
	357.08	0.1277	H-1 → L+1 (−0.36323), H → L+3 (0.59044)	
	355.07	0.3012	H-1 → L+1 (0.64406), H → L (−0.14195), H → L+2 (0.20536), H → L+5 (−0.11757)	
	291.54	0.1378	H-1 → L+5 (0.56611), H → L+4 (0.26197), H → L+6 (0.24790), H → L+8 (0.16802)	289
**7**	363.05	0.1793	H → L (0.32141), H → L+1 (0.59046), H → L+2 (0.20277)	385
	359.62	0.5263	H → L (0.59860), H → L+1 (−0.35664)	256
	243.74	0.2305	H-4 → L (0.52610), H → L+4 (0.41511), H → L+5 (0.12900)	

*Only transitions with oscillator strength (*f*) greater than 0.1 are listed.
